# Incidence and risk factors of intubation-related vocal cord paralysis after unilateral thyroidectomy

**DOI:** 10.1186/s40981-025-00842-4

**Published:** 2025-12-28

**Authors:** Takumi Harata, Ryosuke Yamamoto, Takashi Fujiwara, Kazuyoshi Ishida

**Affiliations:** 1 Department of Anesthesiology, Kurashiki Central Hospital, Kurashiki, Japan; 2 Department of Anesthesiology, Kameda Medical Center, 929 Higashi-cho, Kamogawa, 296-8602 Japan; 3 Department of Otolaryngology, Kurashiki Central Hospital, Kurashiki, Japan

**Keywords:** Vocal cord paralysis, Thyroidectomy, Intubation, Risk factors, Anesthesia

## Abstract

**Background:**

Vocal cord paralysis (VCP) is a recognized complication of endotracheal intubation, but its true incidence may be underestimated because many asymptomatic cases remain undetected. Previous studies have focused primarily on VCP affecting the surgical side after unilateral thyroidectomy, whereas the non-surgical side has received less attention. The present study determines the incidence and risk factors of intubation-induced VCP on the non-surgical side in patients undergoing hemithyroidectomy.

**Results:**

This retrospective cohort included 339 patients who underwent hemithyroidectomy at a single institution between 2010 and 2018. All patients underwent routine pre- and postoperative laryngeal examinations using flexible endoscopy. The incidence of intubation-induced VCP on the non-surgical side was 2.4% (8/339). Patients who developed VCP had significantly lower body weight and body mass index than those without VCP, suggesting that underweight individuals may be more vulnerable. Most cases were transient, resolving within two months of surgery. No significant associations were identified between age, duration of surgery, endotracheal tube size, or intubation method. All cases of intubation-related VCP occurred in patients anesthetized with total intravenous anesthesia. However, this finding should be interpreted with caution because the retrospective design precludes establishing causality and potential confounders.

**Conclusions:**

Intubation-induced VCP was observed more frequently than previously reported, particularly among underweight patients. Routine perioperative laryngeal assessment enables detection of asymptomatic cases and may help clarify risk factors. Careful preoperative evaluation and vigilant intraoperative management remain essential to reduce the risk of this underrecognized complication.

**Supplementary Information:**

The online version contains supplementary material available at 10.1186/s40981-025-00842-4.

## Background

Endotracheal intubation is a routine procedure performed in various surgical and critical care settings. Although generally considered safe, it carries certain risks, particularly for laryngeal structures. Vocal cord paralysis (VCP) is a less commonly recognized but significant intubation complication. This condition can arise from direct injury to the larynx or damage to the recurrent laryngeal nerve, leading to vocal cord paralysis and, in severe cases, hoarseness, voice loss, or airway obstruction. The incidence of VCP on the surgical side after thyroid lobectomy has been reported as 5.8–9.8% [1–3]. In contrast, the incidence of VCP following elective surgery and intubation ranges from 0.03% to 0.08% [4, 5]. Despite its low frequency, VCP is clinically significant because of its potential to cause vocal disability and impaired swallowing. In severe cases, a tracheostomy may be necessary for bilateral paralysis.

The etiology of intubation-related VCP is multifactorial, with the most widely accepted cause being compression of the recurrent laryngeal nerve between the endotracheal tube cuff and thyroid cartilage, as well as direct trauma to the laryngeal structures [6, 7]. Several risk factors have been identified, including advanced age, diabetes, hypertension, prolonged intubation, larger endotracheal tube size, high cuff pressure, and neck extension during intubation [4, 6, 8–10].

Although the incidence of VCP following intubation is typically considered low, these estimates may not fully capture the true prevalence of this condition. Studies often rely on symptom-driven evaluations in which vocal cord function is assessed only in patients exhibiting symptoms such as hoarseness or voice changes. However, this approach may underestimate the incidence of missing subclinical or asymptomatic cases. Professional guidance recommends systematic voice assessment and visualization of vocal fold movement peri-thyroidectomy, supporting routine laryngoscopy rather than symptom-triggered checks [11]. Campbell et al. found a 7% incidence of unilateral vocal fold immobility in a cohort of patients intubated for > 12 h in a medical ICU [8], suggesting that the prevalence of intubation-related VCP may be higher than previously recognized. The study also highlighted that patients with underlying vascular conditions or hypotension requiring vasopressors are at higher risk, supporting the hypothesis that ischemic injury is a potential mechanism [8].

Most studies examining VCP after thyroid surgery have focused on the surgical side, where direct nerve traction or thermal injury is the primary mechanism of dysfunction. In contrast, paralysis occurring on the non-surgical side is less frequently discussed, although endotracheal intubation may contribute to recurrent laryngeal nerve impairment. Because many postoperative evaluations are symptom-driven, asymptomatic or mild non-surgical side injuries may remain unrecognized. We hypothesized that the incidence of intubation-related VCP on the non-surgical side may be higher than that previously reported when routine pre- and postoperative laryngeal examinations are performed. The primary outcome of this study was the incidence of non-surgical-side VCP attributable to endotracheal intubation in patients undergoing hemithyroidectomy. Secondary outcomes included the identification of patient-, surgical-, and anesthesia-related factors associated with VCP development.

## Methods

### Study design

This retrospective cohort study was conducted at Kurashiki Central Hospital and included patients who underwent hemithyroidectomy between April 2010 and March 2018. This study investigated the incidence and risk factors associated with intubation-induced VCP on the non-surgical side following hemithyroidectomy.

### Patient selection

The study population comprised 339 patients who underwent thyroid lobectomy. Patients who underwent total thyroidectomy were excluded to avoid confounding the results with VCP caused by surgical manipulations. Other exclusion criteria included a history of neck surgery or preexisting vocal cord dysfunction.

### Data collection

Data were extracted from the medical records, including patient demographics (age, sex, body weight, and body mass index [BMI]), surgical details, and anesthetic methods used. The specific factors analyzed included the size of the endotracheal tube, the number of intubation attempts, and the intubation method. At our institution, the otolaryngology department has established routine preoperative and postoperative fiberoptic laryngeal examinations for patients undergoing thyroid surgery as usual clinical practice. Preoperative and postoperative nasoendoscopies were performed by otolaryngologists to assess vocal cord function. Therefore, the present study used retrospectively collected data from this prospective clinical database, and all perioperative procedures reflected standard practice during the study period. Postoperative nasoendoscopic examinations were performed either in the operating room immediately after extubation or in the ward on the same day, with follow-up evaluations at one week, and one and two months postoperatively to monitor vocal cord mobility. Intubation-related VCPs were defined as any degree of vocal cord immobility on the contralateral side during surgery.

### Anesthetic procedure

All procedures were performed under general anesthesia. Endotracheal intubation was performed by various healthcare providers, including junior and senior residents, attending physicians, and paramedics. Propofol was used for total intravenous anesthesia (TIVA), and inhalation anesthesia was administered using sevoflurane or desflurane. The choice between TIVA and inhalational anesthesia was made by the attending anesthesiologist, mostly based on the risk of postoperative nausea and vomiting. Rocuronium was administered to all patients, and additional doses were administered at the discretion of the anesthesiologist. Continuous neuromuscular monitoring was not routinely performed. Following intubation, the endotracheal tube cuff was inflated, and the volume of air used was recorded. Cuff pressure was closely monitored, aiming for 25 cmH2O, with precautions to keep it below 30 cmH2O.

### Surgical procedure

In Japan, hemithyroidectomy is the preferred surgical treatment for thyroid conditions over total thyroidectomy. All surgeries in this study were performed using traditional open techniques, with no endoscopic methods used. The procedures were performed exclusively using electrocautery and bipolar forceps, without the use of energy devices such as harmonic scalpels or other advanced surgical tools. Given that this study was conducted at an educational hospital, all patients underwent preoperative and postoperative laryngeal fiberoptic examinations to assess for VCP. Intraoperative nerve monitoring (IONM) was not performed in any patient.

### Statistical analysis

The incidence of intubation-induced VCP was determined, and the cohort was divided into two groups: those with and without VCP. Continuous variables were compared using the Mann–Whitney U test, as none satisfied the normality assumption. Categorical variables were compared using Fisher’s exact test for two categories and the chi-square test for more than two categories. Statistical significance was set at *P* < 0.05. Data analysis was conducted using Python 3.11 and SciPy 1.14.1.

## Results

### Incidence of VCP and patient demographics and clinical characteristics

A total of 339 patients who underwent hemithyroidectomy were included in this study. The overall incidence of VCP on the non-surgical side was 2.4% (8/339). Among these, six patients experienced resolution of VCP within one week, while the remaining two patients recovered fully within two months. Additionally, among the 339 patients, transient VCP on the surgical side was observed in 26 cases (7.7%), and permanent VCP on the surgical side was observed in seven cases (2.1%).

Table 1 summarizes patient characteristics stratified by VCP status. All eight patients who developed VCP were female, although the difference was not significant (*P* = 0.12). Patients who developed VCP had significantly lower body weight (50.0 kg vs. 58.7 kg, *P* < 0.01) and BMI (20.1 vs. 23.4, *P* < 0.01) than those without VCP. There was no significant difference in age between patients with and without VCP (55.5 vs. 60.0 years, *P* = 0.14). No significant differences were observed in terms of pathology, surgical side, diabetes mellitus, hypertension, smoking, or drinking history between the two groups.

**Table 1:** Patient characteristics stratified by the presence of vocal cord paralysis (VCP)

		Overall	VCP -	VCP +	*P*-Value
n		339	331	8	
Gender	Female	249 (73.5)	241 (72.8)	8 (100.0)	0.12
Height (cm)		158.0 [153.0, 165.0]	158.0 [153.2, 165.2]	155.6 [150.8, 158.1]	0.13
Weight (kg)		58.0 [51.2, 66.2]	58.7 [51.3, 66.5]	50.0 [43.5, 51.9]	< 0.01 *
BMI		23.3 [20.9, 25.8]	23.4 [21.0, 26.0]	20.1 [18.7, 21.8]	< 0.01 *
Age		60.0 [48.0, 68.0]	60.0 [48.0, 68.5]	55.5 [40.8, 58.2]	0.14
Pathology	Malignant	252 (74.8)	245 (74.5)	7 (87.5)	0.68
Surgical Side	Right	185 (54.6)	180 (54.4)	5 (62.5)	0.73
Diabetes mellitus	Yes	36 (10.6)	36 (10.9)	0 (0)	1
Hypertension	Yes	112 (33.0)	110 (33.2)	2 (25.0)	1
Cardiovascular disease	Yes	28 (8.3)	28 (8.5)	0 (0)	1
Smoking History	Yes	79 (23.4)	79 (23.9)	0 (0)	0.21
Drinking History	Yes	88 (26.1)	88 (26.7)	0 (0)	0.12

Continuous variables are presented as medians [interquartile ranges], and categorical variables are presented as numbers (percentages). Asterisks (*) denote significant *p*-values. VCP +: patients with vocal cord paralysis on the non-surgical side; *BMI* Body Mass Index

### Surgical and anesthetic factors

Surgical and anesthetic factors are listed in Table 2. The median surgical duration was 117 min (range, 88–142 min). The median surgery time did not differ significantly between the VCP + and VCP- groups (115.0 vs. 117.0 min, *P* = 0.66). No significant differences were found in the tube size or type (reinforced vs. normal) between the groups. The intubation methods (direct laryngoscopy, video laryngoscopy, airway scope, fiberoptic intubation, or laryngeal mask airway) did not differ significantly between groups. Both cuff volume and tube depth were comparable between the VCP + and VCP- groups (*P* = 0.47 and *P* = 0.48, respectively), although the cuff volume could be adjusted to maintain a target pressure of 25 cmH2O. The number of intubation attempts did not differ significantly between groups (*P* = 0.82). A significant association was found between TIVA use and VCP (100% in VCP + vs. 53.5% in VCP-, *P* < 0.01). As expected, due to the nature of TIVA, the total amount of propofol administered was significantly higher in the VCP + group compared to the VCP- group (16.0 mg/kg vs. 9.7 mg/kg, *P* = 0.01). Similarly, the fentanyl dosage was also significantly higher in the VCP + group (5.9 µg/kg vs. 4.0 µg/kg, *P* < 0.01), likely reflecting the need for greater analgesia with TIVA, as volatile anesthetics inherently provide some analgesic effects. There were no cases of temporary or permanent VCP on the surgical side in the VCP + group. The total rocuronium dose did not differ significantly between groups (1.5 mg/kg vs. 1.3 mg/kg, *P* = 0.14).

**Table 2:** Surgical and anesthetic characteristics stratified by the presence of vocal cord paralysis (VCP)

		Overall	VCP -	VCP +	*P*-Value
n		339	331	8	
Surgery Time (min)		117.0 [92.5, 141.5]	117.0 [93.0, 142.0]	115.0 [88.5, 129.5]	0.66
Tube Size (Inner Diameter, mm)		7.0 [7.0, 8.0]	7.0 [7.0, 8.0]	7.0 [7.0, 7.0]	0.12
Tube Type	Reinforced	298 (87.9)	290 (87.6)	8 (100)	1
	Normal	27 (7.9)	27 (8.1)	0 (0)	
Intubation Method	Direct Laryngoscopy	273 (83.7)	267 (84.0)	6 (75.0)	0.88
	Video Laryngoscopy	33 (10.1)	32 (10.1)	1 (12.5)	
	AWS	16 (4.9)	15 (4.7)	1 (12.5)	
	Fiberoptic Intubation	3 (0.9)	3 (0.9)	0 (0)	
	LMA	1 (0.3)	1 (0.3)	0 (0)	
Tube Depth		22.0 [21.0, 23.0]	22.0 [21.0, 23.0]	22.0 [21.0, 22.0]	0.48
Cuff Volume (ml)		5.0 [4.0, 6.0]	5.0 [4.0, 6.0]	5.0 [4.0, 5.0]	0.47
Intubation Attempts		1.0 [1.0, 1.0]	1.0 [1.0, 1.0]	1.0 [1.0, 1.2]	0.82
Anesthesia Method	TIVA	185 (54.6)	177 (53.5)	8 (100.0)	< 0.01 *
	Volatile Anesthesia	154 (45.4)	154 (46.5)		
Infusion Volume (ml/kg)		19.6 [15.2, 26.2]	19.5 [15.1, 26.1]	21.4 [16.9, 27.1]	0.61
Fentanyl (mcg/kg)		4.1 [3.3, 5.1]	4.0 [3.3, 4.9]	5.9 [5.8, 6.5]	< 0.01 *
Propofol (mg/kg)		10.5 [1.4, 15.8]	9.7 [1.4, 15.7]	16.0 [15.3, 17.0]	0.01 *
Rocuronium (mg/kg)		1.3 [1.1, 1.5]	1.3 [1.1, 1.5]	1.5 [1.3, 1.6]	0.14
Norepinephrine (mcg/kg)		0.0 [0.0, 0.0]	0.0 [0.0, 0.0]	0.0 [0.0, 0.0]	0.94
Phenylephrine (mcg/kg)		0.0 [0.0, 0.0]	0.0 [0.0, 0.0]	0.0 [0.0, 0.0]	0.71
Ephedrine (mg/kg)		0.1 [0.0, 0.2]	0.1 [0.0, 0.2]	0.0 [0.0, 0.1]	0.37
Temporary VCP On Surgical Side	Yes	26	26 (7.9)	0 (0)	1
Permanent VCP On Surgical Side	Yes	7	7 (2.1)	0 (0)	1

Continuous variables are presented as medians [interquartile ranges], and categorical variables are presented as numbers (percentages). Asterisks (*) denote significant p-values. VCP +, patients with vocal cord paralysis on the non-surgical side; *TIVA* Total Intravenous Anesthesia, *AWS* Airway Scope, *LMA* Laryngeal Mask Airway

## Discussion

This study investigated the incidence and risk factors for intubation-induced VCP in patients undergoing thyroid lobectomy. The incidence of VCP on the non-surgical side was 2.4%, which was significantly higher than the reported incidence for general surgeries not involving the thyroid, which ranges from 0.03% to 0.08% [4, 5]. This discrepancy may be attributed to the comprehensive nature of our study, as all patients underwent nasoendoscopy performed by an otolaryngologist, enabling detection of transient or mild cases of VCP. Furthermore, this may be partly explained by the nature of thyroid surgery, in which neck manipulation can increase pressure between the tracheal tube and the thyroid cartilage, potentially damaging the recurrent laryngeal nerve on the non-surgical side. Significantly, all eight patients with intubation-related VCP fully recovered, consistent with previous studies where intubation-related VCP was rare (0.03–0.08%). Additionally, although all surgeries in our study were completed in less than three hours, the incidence of VCP may be even higher in longer procedures.

### Risk factors

Our analysis identified several factors associated with an increased risk of intubation-induced VCP. Notably, although all VCP cases occurred in female patients, the difference was not significant, likely because of the predominance of females in the study population. By contrast, both body weight and BMI were significantly lower in patients with VCP, indicating that underweight individuals may be particularly vulnerable. Reduced soft-tissue buffering around the recurrent laryngeal nerve may render these patients more susceptible to compression during intubation and neck manipulation, a mechanism supported by previous anatomical studies [6, 7]. Low BMI has also been reported as a risk factor for arytenoid cartilage dislocation, another cause of postoperative vocal-fold immobility, and several authors have suggested that underweight patients may be predisposed to mechanical injury of the cricoarytenoid joint [10, 12]. However, in our cohort, all the patients with intubation-related VCP recovered fully and spontaneously. As spontaneous recovery is uncommon in true arytenoid dislocations, this pattern suggests that most cases in this study were more consistent with transient neuropraxia of the recurrent laryngeal nerve rather than with mechanical displacement of the arytenoid.

In contrast, other commonly reported risk factors, such as age, diabetes, hypertension, cardiovascular disease, and tube size, did not show a significant effect in our study [4, 8, 9]. These variables have primarily been identified in critically ill or prolonged intubation populations, in which ischemic mechanisms related to atherosclerosis and malperfusion are more prominent. Our cohort consisted of generally healthy patients who underwent short elective thyroid surgery, resulting in limited variability in vascular comorbidities and minimal vasopressor use. Although previous studies have suggested that hypotension and vasopressor requirement may contribute to laryngeal injury, vasopressor dosing in our population was low. It showed no significant association with VCP, and intraoperative blood pressure data were not available in a structured format suitable for retrospective analysis.

### Mechanisms of intubation-induced VCP

Classic endoscopic data show that rising cuff pressure compromises mucosal perfusion, supporting vigilant manometry and avoiding excessive pressures [13]. Although Taylor et al. demonstrated that increased cuff pressure can significantly alter the conductivity of the recurrent laryngeal nerve during thyroid surgery [6], our findings did not allow us to draw definitive conclusions about the impact of cuff pressure. However, it is plausible that elevated cuff pressure during surgery, especially in procedures requiring neck extension or thyroid gland manipulation, contributed to the observed cases of VCP. Although we recorded cuff air volume injected immediately after intubation and confirmed that cuff pressure did not exceed 30 cmH2O, we did not continuously monitor cuff pressure throughout the surgery. Consequently, cuff pressure is an important consideration; its precise role in our cohort cannot be overlooked. The cuff position also matters; placing the cuff at least 15 mm below the vocal folds (and below the cricoid) may reduce focal pressure on the recurrent laryngeal nerve during surgical positioning [14]. Conceptually, a mismatch between endotracheal tube diameter and individual laryngeal dimensions could increase VCP risk [4, 15]; however, within the routinely used sizes in this cohort (men: 7.5–8.0 mm; women: 6.5–7.0 mm), we observed no association, with limited power to detect minor effects.

### VCP on the surgical side

In our study, transient VCP related to the surgical side was observed in 26 patients (7.7%), which is comparable to the previously reported incidence of 8.3% [1]. Although VCP on the surgical side is typically attributed to direct surgical manipulation, the incidence of intubation-related VCP on the non-surgical side suggests that similar mechanisms could also occur on the surgical side. Therefore, efforts to reduce intubation-related VCP may mitigate the risk of VCP during surgery, highlighting the importance of comprehensive prevention strategies.

### TIVA vs. Volatile anesthesia

In our study, all cases of VCP occurred in patients who were anesthetized with propofol. TIVA with propofol may result in less muscle relaxation than with volatile anesthesia [16]. Although rocuronium dosing did not differ between groups, similar dosing may represent relatively insufficient relaxation in the TIVA group. This relative relaxation shortage may increase laryngeal muscle tension, potentially heightening recurrent laryngeal nerve compression during surgical neck manipulation. These findings should be interpreted cautiously; however, the pattern is consistent with the known pharmacological differences between anesthetic techniques.

### Clinical implications

The incidence of intubation-related vocal-fold immobility observed in this study was higher than generally reported, indicating that such injuries may be underrecognized when routine laryngeal examinations are not performed. This underscores the importance of adopting perioperative strategies that may help reduce the occurrence of these events. Although low BMI was the only significant risk factor identified, several practical measures are reasonable, including frequent monitoring of cuff pressure during neck manipulation, avoidance of excessive neck extension during positioning, and careful assessment of vocal-fold mobility after extubation, particularly in underweight patients. In addition, the finding that VCP occurs exclusively in patients undergoing TIVA warrants further investigations. Volatile anesthetics potentiate neuromuscular blockade, whereas TIVA does not; however, the precise mechanism underlying the higher VCP incidence in the TIVA group remains to be elucidated. Prospective studies with standardized neuromuscular monitoring may clarify whether differences in muscle relaxation, endotracheal tube dynamics, or other factors contribute to this association. Overall, our findings highlight the need for heightened vigilance during thyroid surgery and suggest that targeted attention to airway management in vulnerable patients may help reduce the risk of postoperative vocal-fold immobility.

### Limitations

This study has some limitations. As a retrospective analysis, it was subjected to potential bias related to data accuracy and completeness. The lack of continuous cuff pressure measurements limits our ability to identify a possible causal link between cuff pressure and VCP. Additionally, specific examinations were not performed to distinguish between direct vocal cord injury and nerve injury, which could have provided further insight into the mechanisms of VCP. The incidence of intubation-related VCP during thyroid surgery has not been extensively reported, and the unexpectedly high rate observed in this study may be linked to indirect surgical factors such as neck extension, tracheal manipulation, and pressure on the thyroid cartilage. Additionally, the timing of nasolaryngoscopy, whether performed in the operating room or ward, has not been consistently documented. Consequently, the potential impact of timing on VCP incidence could not be evaluated. The absence of IONM was a limitation of this study. Although IONM has become increasingly common, current guidelines describe its use as optional, noting that it may be performed during thyroidectomy for malignancy and is particularly recommended for total or reoperative surgery (conditional recommendation, low–moderate certainty). Hemithyroidectomy was the standard approach for our population, and IONM was not routinely implemented for unilateral procedures at our institution during the study period. Moreover, the non-surgical recurrent laryngeal nerve is not exposed during a typical hemithyroidectomy; therefore, IONM does not reliably detect functional changes on the contralateral side. Nevertheless, its absence limits our ability to distinguish subtle intraoperative changes in the surgical-side nerve conductivity.

## Conclusions

This study revealed a higher-than-expected incidence of intubation-induced VCP (2.4%). Importantly, the true incidence of subclinical VCP may be higher than reported, as meticulous screening in our study likely captured cases that might otherwise have gone undetected. Low body weight and BMI were identified as significant risk factors for VCP, suggesting that underweight individuals are more vulnerable to VCP. This study suggests a possible association between propofol-based TIVA and the development of VCP during thyroidectomy. These findings emphasize the need for careful intraoperative management to reduce the risk of VCP, particularly in vulnerable patients, and call for further research to understand the contributing factors better.

## Supplementary Information

Supplementary Material 1.

## Data Availability

Not applicable.

## Abbreviations

AWS: Airway Scope
BMI: Body Mass Index
IONM: Intraoperative Nerve Monitoring, IRB: Institutional Review Board
LMA: Laryngeal Mask Airway
TIVA: Total Intravenous Anesthesia
VCP: Vocal Cord Paralysis

## References

[1] Moreira A, Forrest E, Lee JC, Paul E, Yeung M, Grodski S, et al. Investigation of recurrent laryngeal palsy rates for potential associations during thyroidectomy. ANZ J Surg. 2020;90:1733–7. 10.1111/ans.16166. 32783252 10.1111/ans.16166

[2] Heikkinen M, Mäkinen K, Penttilä E, Qvarnström M, Kemppainen T, Löppönen H, et al. Incidence, risk factors, and natural outcome of vocal fold paresis in 920 thyroid operations with routine pre- and postoperative laryngoscopic evaluation. World J Surg. 2019;43:2228–34. 10.1007/s00268-019-05021-y. 31065775 10.1007/s00268-019-05021-y

[3] Schneider R, Randolph GW, Dionigi G, Wu CW, Barczynski M, Chiang FY, et al. International neural monitoring study group guideline 2018 part I: staging bilateral thyroid surgery with monitoring loss of signal. Laryngoscope [Internet]. 2018. 10.1002/lary.27359. 30289983 10.1002/lary.27359

[4] Kikura M, Suzuki K, Itagaki T, Takada T, Sato S. Age and comorbidity as risk factors for vocal cord paralysis associated with tracheal intubation. Br J Anaesth. 2007;98:524–30. 10.1093/bja/aem005. 17341543 10.1093/bja/aem005

[5] Sariego J. Vocal fold hypomobility secondary to elective endotracheal intubation: a general surgeon’s perspective. J Voice. 2010;24:110–2. 10.1016/j.jvoice.2008.05.001. 19185456 10.1016/j.jvoice.2008.05.001

[6] Taylor JW, Soeyland K, Ball C, Lee JC, Serpell J. Changes in tracheal tube cuff pressure and recurrent laryngeal nerve conductivity during thyroid surgery. World J Surg. 2020;44:328–33. 10.1007/s00268-019-05185-7. 31552459 10.1007/s00268-019-05185-7

[7] Apfelbaum RI, Kriskovich MD, Haller JR. On the incidence, cause, and prevention of recurrent laryngeal nerve palsies during anterior cervical spine surgery. Spine (Phila Pa 1976). 2000;25:2906–12. 10.1097/00007632-200011150-00012. 11074678 10.1097/00007632-200011150-00012

[8] Campbell BR, Shinn JR, Kimura KS, Lowery AS, Casey JD, Ely EW, et al. Unilateral vocal fold immobility after prolonged endotracheal intubation. JAMA Otolaryngol Head Neck Surg. 2020;146:160–7. 10.1001/jamaoto.2019.3969. 31855261 10.1001/jamaoto.2019.3969 PMC6990766

[9] Santos PM, Afrassiabi A, Weymuller EA. Risk factors associated with prolonged intubation and laryngeal injury. Otolaryngol Head Neck Surg. 1994;111:453–9. 10.1177/019459989411100411. 7936678 10.1177/019459989411100411

[10] Wang K, Chen R, Zeng Z, Liu min, Meng tao. Hoarseness after general endotracheal anesthesia: a single-center retrospective analysis. J Anesth Transl Med. 2024;3:60–4. 10.1016/j.jatmed.2024.05.002 PMC13001764 41930236

[11] Chandrasekhar SS, Randolph GW, Seidman MD, Rosenfeld RM, Angelos P, Barkmeier-Kraemer J, et al. Clinical practice guideline: improving voice outcomes after thyroid surgery. Otolaryngol Head Neck Surg. 2013;148(Suppl):S1–37. 10.1177/0194599813487301. 23733893 10.1177/0194599813487301

[12] Lou Z, Yu X, Li Y, Duan H, Zhang P, Lin Z. BMI may be the risk factor for arytenoid dislocation caused by endotracheal intubation: a retrospective case-control study. J Voice. 2018;32:221–5. 10.1016/j.jvoice.2017.05.010. 28601417 10.1016/j.jvoice.2017.05.010

[13] Seegobin RD, van Hasselt GLV. Endotracheal cuff pressure and tracheal mucosal blood flow: endoscopic study of effects of four large volume cuffs. Br Med J (Clin Res Ed). 1984;288:965–8. 10.1136/bmj.288.6422.965. 6423162 10.1136/bmj.288.6422.965 PMC1442489

[14] Kim H, Chang JE, Ryu JH, Jung H, Min SW, Lee JM, et al. Retrospective analysis of vocal cord-to-suprasternal notch distance: implications for preventing endotracheal tube cuff-induced vocal cord injury. Medicine. 2017;96:e6155. 10.1097/MD.0000000000006155. 28207550 10.1097/MD.0000000000006155 PMC5319539

[15] Anwar M, Hussain KA, Anwar P. Vocal cord paralysis after tracheal intubation: an overview of the etiology and associated risk factors. Int Arch Otorhinolaryngol. 2025;29:1–7. 10.1055/s-0045-1808244. 40625534 10.1055/s-0045-1808244 PMC12234165

[16] Suzuki T, Munakata K, Watanabe N, Katsumata N, Saeki S, Ogawa S. Augmentation of vecuronium-induced neuromuscular block during sevoflurane anaesthesia: comparison with balanced anaesthesia using propofol or midazolam. Br J Anaesth. 1999;83:485–7. 10.1093/bja/83.3.485. 10655928 10.1093/bja/83.3.485

