# Supplementary material for: Incidence and risk factors of intubation-related vocal cord paralysis after unilateral thyroidectomy
Source: JA Clin Rep. 2025 Dec 28;12:11. doi: 10.1186/s40981-025-00842-4 (PMC12855656; doi:10.1186/s40981-025-00842-4)
Supplement: Supplementary file 1 — Supplementary Material 1. [file 40981_2025_842_MOESM1_ESM.docx]

**Rebuttal Letter for Manuscript: JARP-D-25-00267R1**

We sincerely thank you and the reviewers for evaluating our manuscript and for the constructive comments provided. We are grateful for the indication that the revised manuscript is potentially acceptable for publication. We have addressed the remaining point raised by Reviewer #2 and made the corresponding correction.

Below, we provide our response. All revisions have been highlighted in **red** in the revised manuscript, and reference numbers have been updated accordingly.

# **Response to Reviewer Comments**

### **Reviewer #1**

The questions and points for improvement raised by the reviewers were appropriately addressed in the new manuscript.

**Response:**
We thank the reviewer for the positive assessment and have no further changes in response to this comment.

### **Reviewer #2 — Minor Comment**

**Comment:**
P10 L25:

*“Given that volatile anesthetics potentiate…”
This sentence should be removed because this point should be addressed in the Discussion section rather than in the Results section.*

**Response:**
Thank you for this careful observation. We agree that this mechanistic interpretation belongs in the Discussion rather than the Results. Accordingly, we have **removed the sentence from the Results section**, and the reference numbers have been renumbered to reflect this change.

We appreciate the opportunity to revise our work and thank the editorial team once again for their guidance. We hope that the revised manuscript is now suitable for publication in JA Clinical Reports.

Sincerely,
**Ryosuke Yamamoto, MD. PhD.**
